# Unified wavelet and gaussian filtering for segmentation of CT images; application in segmentation of bone in pelvic CT images

**DOI:** 10.1186/1472-6947-9-S1-S8

**Published:** 2009-11-03

**Authors:** Simina Vasilache, Kevin Ward, Charles Cockrell, Jonathan Ha, Kayvan Najarian

**Affiliations:** 1 Department of Computer Science, Virginia Commonwealth University, Richmond, VA, USA; 2 Emergency Department, School of Medicine, Virginia Commonwealth University, Richmond, VA, USA; 3 Radiology Department, School of Medicine, Virginia Commonwealth University, Richmond, VA, USA

## Abstract

**Background:**

The analysis of pelvic CT scans is a crucial step for detecting and assessing the severity of Traumatic Pelvic Injuries. Automating the processing of pelvic CT scans could impact decision accuracy, decrease the time for decision making, and reduce health care cost. This paper discusses a method to automate the segmentation of bone from pelvic CT images. Accurate segmentation of bone is very important for developing an automated assisted-decision support system for Traumatic Pelvic Injury diagnosis and treatment.

**Methods:**

The automated method for pelvic CT bone segmentation is a hierarchical approach that combines filtering and histogram equalization, for image enhancement, wavelet analysis and automated seeded region growing. Initial results of segmentation are used to identify the region where bone is present and to target histogram equalization towards the specific area. Speckle Reducing Anisotropic Didffusion (SRAD) filter is applied to accentuate the desired features in the region. Automated seeded region growing is performed to refine the initial bone segmentation results.

**Results:**

The proposed method automatically processes pelvic CT images and produces accurate segmentation. Bone connectivity is achieved and the contours and sizes of bones are true to the actual contour and size displayed in the original image. Results are promising and show great potential for fracture detection and assessing hemorrhage presence and severity.

**Conclusion:**

Preliminary experimental results of the automated method show accurate bone segmentation. The novelty of the method lies in the unique hierarchical combination of image enhancement and segmentation methods that aims at maximizing the advantages of the combined algorithms. The proposed method has the following advantages: it produces accurate bone segmentation with maintaining bone contour and size true to the original image and is suitable for automated bone segmentation from pelvic CT images.

## Introduction

Traumatic injuries are leading cause of death for patients with ages up to 45 years. Every year, on U.S territory alone, a staggering total of four million years of potential life are cut short due to traumatic injuries [1]. Forty percent of the patients with fatal traumatic injuries die before even reaching the emergency room [2]. Motor vehicle crashes account for 48% to 68% of traumatic injuries. Among traumatic injuries, Traumatic Pelvic Injury and associated complications, such as internal hemorrhage, infected hematomas, multi-organ failure and blood clots traveling to the brain, result in a mortality rate ranging from 8.6% to 50% [3]. Even when the injury is not fatal it is usually the cause of life long disabilities [4]. Computer-aided systems can impact trauma decision making by increasing decision accuracy and reducing time for decision making. Such effects can, in turn, lead to improved health care standards, better resource allocation and lower health care costs.

Traumatic injury decision making is extremely time sensitive therefore a decrease in decision making time is extremely significant for patient treatment. Because traumatic injuries are associated with specific causes and treatment methods, the chance of occurrence for fatality or long-term disability can be avoided or reduced by making more accurate decisions in the trauma unit [5]. Although several computer-assisted trauma decision making systems already exist, the majority of the systems rely solely on patient demographics to find similar cases in trauma databases and provide a recommendation based on these cases. As a result the recommendations might not be accurate or specific enough for the purpose of practical implementation [5].

Detecting the presence and extent of the fracture is an important step in assessing the severity of a pelvic injury. Subtle fractures, as fractures of the acetabulum, hip displacement or presence of hemorrhage, are assessed based on the analysis of Computed Tomography (CT) images, as CT is more detailed than X-Ray. Since the number of CT slices can be quite large, it is important to develop a computer aided decision making system to analyze the data, because in general only a small portion of the dataset becomes important in establishing a diagnostic [6].

Segmentation of CT images poses a series of typical challenges. The density of cortical bone is significantly different than that of cancellous bone, as a result the appearance and overall grey level of the two types of bone tissue are very different. Cortical bone is bright and smooth while cortical bone has a spongy texture and an intensity similar to that of the surrounding tissue. It is for this reason that the bone density cannot be uniformly characterized [7]. Additionally, CT scans typically includes tens of slices in which bones assume different shapes and positions depending on the patient vertical and horizontal alignment during the scanning procedure. A number of slices include joint regions where the normal distance between bones is very small. Such small distances can result in separate bones erroneously being merged with each other during segmentation.

In this paper, an automated method to segment bone from pelvic CT images is presented. Another portion of our work addresses hemorrhage detection in pelvic CT images using advanced image processing techniques. The following paragraphs will briefly discuss several existing segmentation techniques used for medical image segmentation.

X-ray computed tomography (CT) images are typically affected by partial volume effects which cause the transitions from bone to air or bone to fat to be less noticeable [8]. Such faded transitions result in similar grey level values of bone and surrounding soft tissue. A popular approach in dealing with low contrast images and noisy edges is using methods that are based on deformable models (DMM) [8]. DMMs allow knowledge about adjacent structures to be incorporated in the model and therefore are capable of producing more accurate segmentation [9]. Deformable Model Methods (DMM) [9-13] are parametric segmentation methods that use closed curves or surfaces that can be deformed under internal and external forces. The intrinsic advantage of such methods is directly generating closed curves while some of the disadvantages include poor convergence towards concave boundaries and sensitivity to initialization. Another category of popular segmentation techniques are based on watershed transform. Watershed segmentation techniques [6,14-17] are gradient based methods that the image based on its topology. Watershed methods can often result in over-segmentation of the image. Approaches to minimize over-segmentation include: marker-based watershed and low pass filtering of the image prior to watershed segmentation.

Region Growing methods [18-20] are very popular for the segmentation of medical images. In their classic form such methods segment an image by iteratively adding neighboring pixels, that satisfy certain similarity constraints, to the initial seeds. The major advantages of Region Growing are the simple concept, implementation and possibility of using multiple similarity criteria. Region Growing methods share the common disadvantage of being dependent on initialization - quality of segmentation is dependent of initialization quality.

Region Competition segmentation [21,22] combines statistical properties of region growing methods with geometrical features of deformable models. Region Competition methods are also dependent on initialization and the precision of boundary detection is dependent on the size of the sampling window as well as the signal to noise ratio.

Level Set Methods (LSM) [23-26] are based on defining a moving contour as the zero-level set of a time-evolving scalar function defined over a regular grid. The curve is deformed according to the solution given by a set of partial differential equations (PDEs). As to how the initial curve can evolve, there are several different approaches: some apply a global update of the function while others are based on local update techniques.

## Methods

### Methodology overview

**Figure 1:**
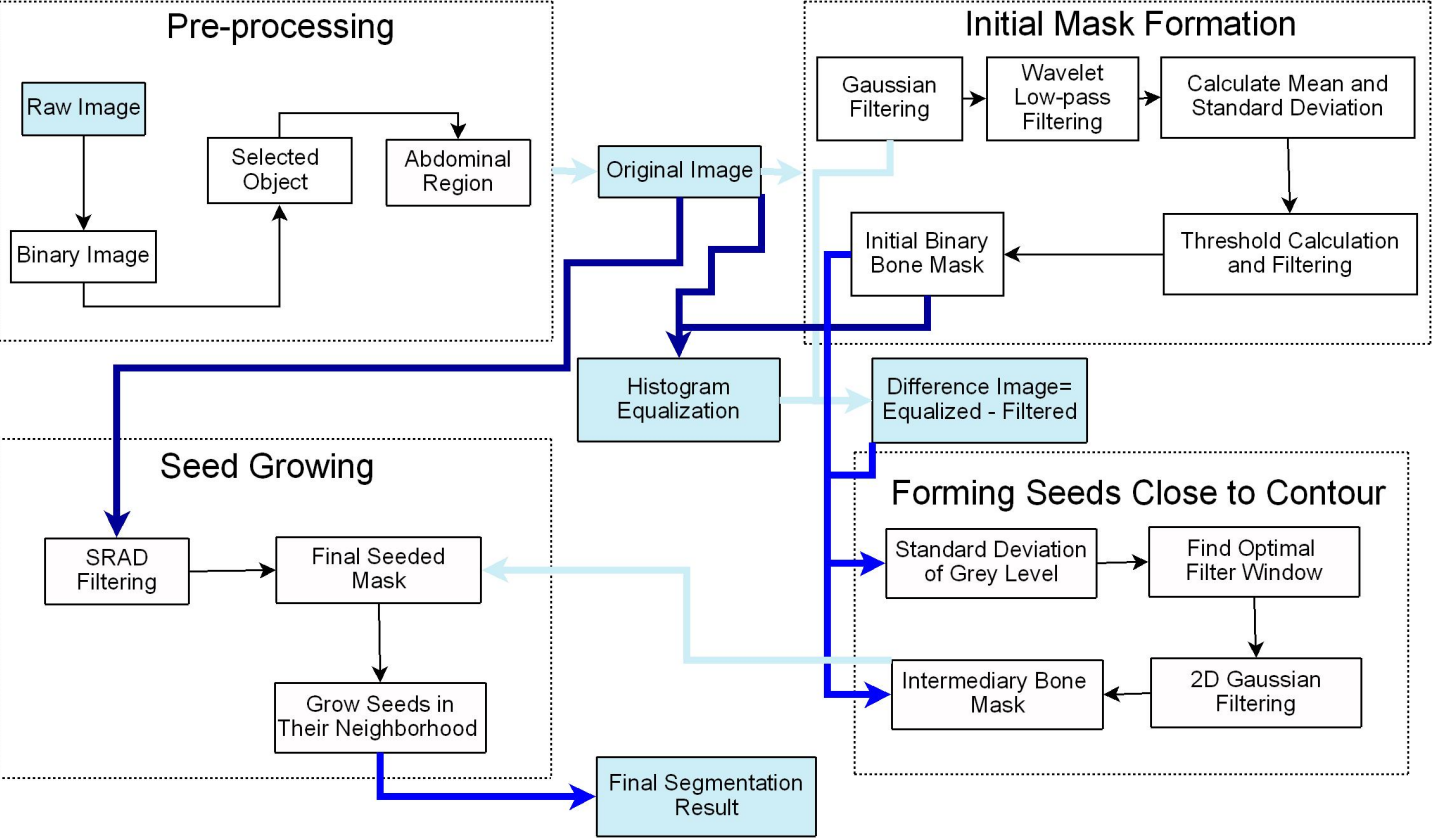
**Schematic diagram for the bone segmentation method**. The schematic diagram of the algorithm includes the major steps of the method grouped into significant stages.

Figure 1 is an overview of the proposed segmentation methodology. The first step is to pre-process the image in order to eliminate artifacts and target segmentation to the abdominal region. We proceed at forming an initial raw segmentation - initial bone mask. This step helps in further constraining the region that is the focus of segmentation. The image region is adjusted using histogram equalization to enhance contrast. The next step is to automatically select seeds that have a high chance of being located close to the edge of the bone segments. Speckle Reducing Anisotropic Diffusion (SRAD) [27] filtering is performed. SRAD is an edge sensitive speckle reducing filter which can enhance the CT images that are subject to segmentation. Automated seed growing is the final step of the proposed segmentation method. More details about the steps presented in the schematic diagram in Figure 1 are provided in the section that follows.

### Pre-processing

The pre-processing step is distinguishing between the abdominal region and the surrounding artifacts, such as the CT table, cabling and lower extremities of the patient that are visible in some images. The segmentation of the abdominal region from the background objects is accomplished by the following process:

1. Create a binary version of the raw image. All the pixels with grey level higher than zero become one. Pixels with grey value zero remain zero in the new image;

2. Apply morphological operations to the binary image so that the different objects in the image are separated;

3. Use blob analysis to select the object that has the largest area;

4. Detect the edge of the object. Create an alternate edge with better symmetry;

5. Create a mask that follows the edge created at step 4.

### Initial mask formation

After eliminating the background from the image, segmentation of bone becomes the focus of the method. From now on the image of the abdomen will be referred to as "the original image". The steps taken to obtain the initial bone segmentation are as follows:

1. Apply a Gaussian filter to the original image;

2. Apply Wavelet analysis to the resulting image. Reconstruct the image using only the approximation matrix;

3. Calculate the means and deviation of the grey scale values that differ from zero, i.e. the pixels that are not background;

4. Calculate threshold value as the sum of means and standard deviation of the grey scale values calculated at Step 3;

Threshold value *t*_1 _is the summation of the modified means *m*_1 _and standard deviation *st*_1 _of the grey values of the pixels that are not background:

(1)

where

(2)

and

(3)

(4)

is the set of background pixels and have zero grey level value. *Card*(*S*) is the notation used for the cardinality of set *S*.

5. Create an initial binary mask for bone by thresholding the low pass image;



6. Determine the bounding box for the bone region. Constrain following segmentation to the identified bounding box;

7. Perform histogram equalization on the image.

### Forming seeds close to contour

The step of the algorithm presented in this section is refining the initial bone mask into an improved intermediary bone mask. A two dimensional Gaussian filter is applied in order to enhance the contours in the image. Below is a brief description of the steps in this stage:

1. Determine the optimal size of the filter window based on *σ *- the standard deviation of the grey level values of the image pixels;

2. Apply an optimal two dimensional Gaussian filter to a copy of the original image;

The resulting image *y*_*g *_pixel values are given by:

(5)

3. Obtain the intermediary bone mask through binary multiplication of the initial bone mask and the Gaussian filtered image:

(6)

### Seed growing

Seed Growing is the final stage of segmentation. In order to automatically obtain the seeded bone mask the intermediary mask is multiplied with a copy of the original image after SRAD filtering. The output image is derived from the original image as follows:



where *I*_*O*_(*x*, *y*; *t*) is the original image, *I*(*x*, *y*; *t*) is the output image, Ω is the image support, ∂Ω is the border of Ω,  is the outer normal to ∂Ω and *c*(*q*) is the instantaneous coefficient of variation.

The Speckle Reducing Anisotropic Diffusion (SRAD) filter is further described in [27] and will reduce the speckle effect in the CT image while preserving the edges. Good segmentation results are produced by growing the seeds from the seeded bone mask in their neighborhood. The region growing technique that was used is described below:

1. For each seed in the seeded bone mask the neighbors in an *n *× *n *neighborhood are identified (in this study *n *= 3);

2. For each seed in the seeded bone mask the values of the gradient along the eight possible directions are calculated in the *n *× *n *neighborhood;

3. For each of the neighbors in the nxn neighborhood their *mxm *neighborhood is identified (in this study *m *= 9);

4. Average grey level value of the neighbors in the mxm neighborhood is calculated;

5. A decision of adding a neighbor of a seed to the seeded bone mask is made based on two conditions:

(a) The neighbor grey level value is greater than the average grey level value of the neighbors in the *m *× *m *neighborhood;

(b) the gradient value corresponding to the neighbor is greater or equal to -1. This is a conservative criteria is meant to keep the seeds from growing outside of the edges of the bone and minimizes the risk of separate bones being merged.

The results provided by the proposed method will be discussed in what follows.

## Results

### Data

The testing CT dataset is courtesy of the Carolinas Healthcare System (CHS). CT scans pertain to Traumatic Pelvic Injury patients. The testing dataset consists of approximately 200 axial pelvic CT images from different segments of the CT scan.

### Results of the proposed method

**Figure 2:**
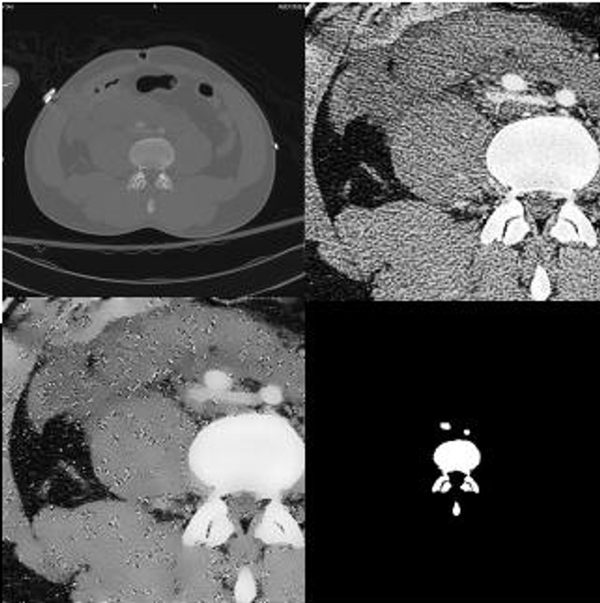
**Sample Result**. The original image is in the upper left corner. The image in the upper right corner is the image after cropping it to the region in which bone is found and histogram equalization. In the lower left corner is the image after Speckle Reducing Anisotropic Diffusion (SRAD) filtering. In the lower right corner are results of segmentation. It can be noticed that the detected bone contour and shape are true to the actual bone contour and size. The separation between bones is maintained even when neighboring bones very close to one another.

The proposed segmentation method was tested on 189 pelvic CT images and in 83% of the images produced acceptable contours. Figures 2 through 7 show sample results of segmentation. Inaccuracy was noticed in the cases where image quality is very poor and where the bones that need to be segmented have an intricate edge and not uniform texture and grey level. However, the algorithm proves to be robust and capable to segment bone from images pertaining to different segments of a CT scan. The size and contour of the identified objects are maintained. In the case of neighboring bones or joint regions the distance is still visible in the segmentation results and adjacent bones are not merged together.

**Figure 3:**
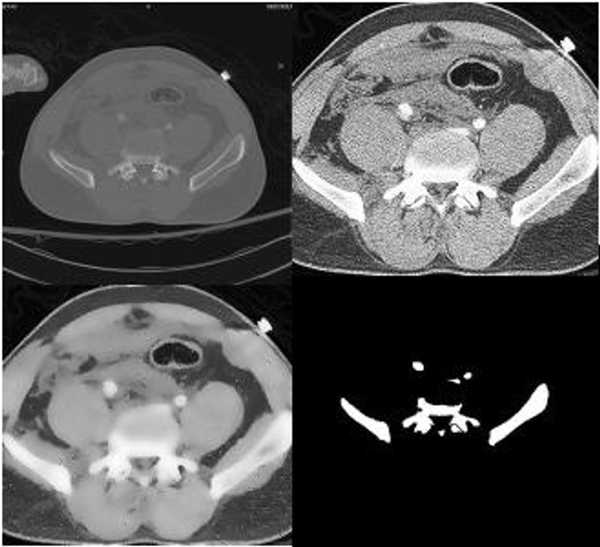
**Sample Result**. The original image is in the upper left corner. The image in the upper right corner is the image after cropping it to the region in which bone is found and histogram equalization. It can be observed that histogram accentuates the appearance of arteries in the center of the image. In the lower left corner is the image after Speckle Reducing Anisotropic Diffusion (SRAD) filtering. In the lower right corner are results of segmentation. The edges of the segmentation results are clearly defined and segmented objects are correctly representing the location, shape and size of the bones visible in the raw image.

**Figure 4:**
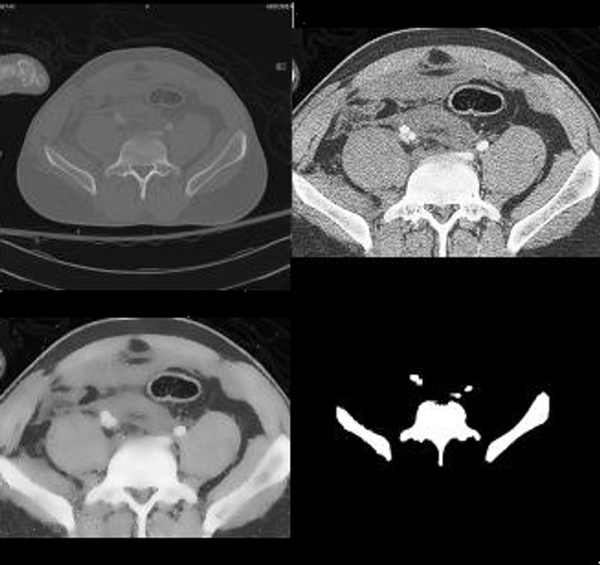
**Sample Result**. The original image is in the upper left corner. The image in the upper right corner is the image after cropping it to the region in which bone is found and histogram equalization. In the lower left corner is the image after Speckle Reducing Anisotropic Diffusion (SRAD) filtering. It can be observed that histogram equalization and SRAD filtering have an impact on the original image. The edges are maintained, contrast is enhanced, and texture is eliminated therefore eliminating some of the challenges in segmentation. In the lower right corner are results of segmentation. It can be noticed that the detected bone contour and shape are true to the actual bone contour and size.

**Figure 5:**
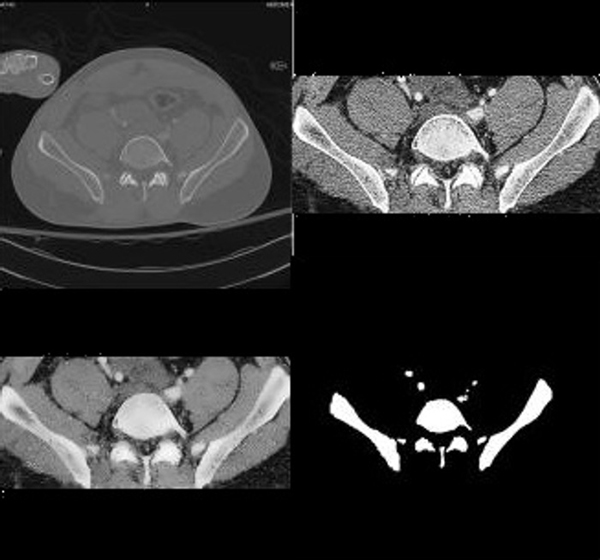
**Sample Result**. The original image is in the upper left corner. The image in the upper right corner is the image after cropping it to the region in which bone is found and histogram equalization. In the lower left corner is the image after Speckle Reducing Anisotropic Diffusion (SRAD) filtering. In the lower right corner are results of segmentation. It can be noticed that the detected bone contour and shape are true to the actual bone contour and size. The method accentuates the appearance of bone regions that are faded in the current slice of the CT scan but more pronounced in the adjacent slices - observe the center region of the segmentation result.

**Figure 6:**
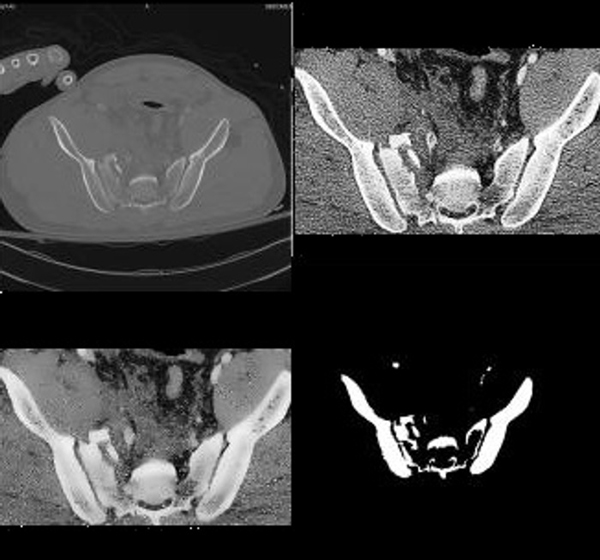
**Sample Result**. The original image is in the upper left corner. The image in the upper right corner is the image after cropping it to the region in which bone is found and histogram equalization. In the lower left corner is the image after Speckle Reducing Anisotropic Diffusion (SRAD) filtering. In the lower right corner are results of segmentation. This is an example in which the proposed method fails to provide accurate segmentation results. The segmentation of the bone in the center of the image is very challenging. The bone's texture and gray level is not uniform and the edge is very intricate: the edge of the respective bone is frequently changing direction at sharp angles - these to factors result in poor segmentation.

**Figure 7:**
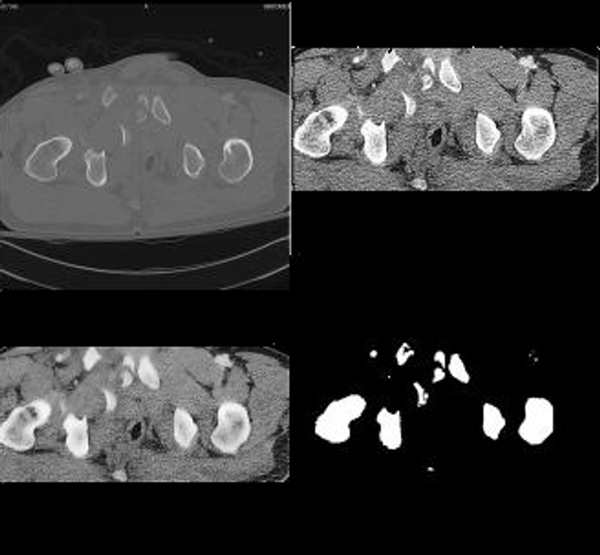
**Sample Result**. The original image is in the upper left corner. The image in the upper right corner is the image after cropping it to the region in which bone is found and histogram equalization. In the lower left corner is the image after Speckle Reducing Anisotropic Diffusion (SRAD) filtering. In the lower right corner are results of segmentation. Results of the proposed method are accurate and faithful to original bone contours. This particular image is an example of an image which would be very difficult to segment using an atlas based approach due to high fragmentation of the bone.

The algorithm has an approximate processing time of 30 seconds for each CT image, from initial input of the image to providing segmentation results. Such a performance suggests a considerable reduction in processing time compared to semi-automated segmentation methods. Semi-automated segmentation methods often require manual selection of seeds and many times such selection is performed in a repetitive process to ensure adequacy of seeds.

## Conclusion and future work

The paper is describing a method for automatic bone segmentation from pelvic CT images. Accurately segmenting CT pelvic images in an automatic manner is an important initial step for assessing the presence of fracture in the context of an assisted decision-making system that provides recommendations for trauma care. The system will integrate demographic information, physiological data, fracture detection, assesment of hemorrhage severity to provide caregivers with recommendations regarding patient diagnosis and treatment. Image analysis for hemorhage detection already provided promising results. A larger database has recently been made available for testing future work includes even more extensive testing of the method.

## List of abbreviations used

(SRAD): Speckle Reducing Anisotropic Difusion; (CT): Computed Tomography; (DMM): Deformable Model Methods; (LSM): Level Set Methods; (PDEs): partial diferential equations

## Competing interests

The authors declare that they have no competing interests.

## Authors' contributions

SV contributed in the design and implementation of the algorithms, segmentation result comparison and drafting the manuscript. KW, CC and JH participated in assesing the results. KN participated in design of the algorithms and revision of manuscript.
